# Estimation of the reproduction number and early prediction of the COVID-19 outbreak in India using a statistical computing approach

**DOI:** 10.4178/epih.e2020028

**Published:** 2020-05-09

**Authors:** Karthick Kanagarathinam, Kavaskar Sekar

**Affiliations:** 1 Department of EEE, GMR Institute of Technology, Rajam, India; 2 Department of EEE, Panimalar Engineering College, Chennai, Tamilnadu, India

**Keywords:** Basic reproduction number, COVID-19, Forecasting, Statistical computing

## Abstract

Coronavirus disease 2019 (COVID-19), which causes severe respiratory illness, has become a pandemic. The World Health Organization has declared it a public health crisis of international concern. We developed a susceptible, exposed, infected, recovered (SEIR) model for COVID-19 to show the importance of estimating the reproduction number (R_0_). This work is focused on predicting the COVID-19 outbreak in its early stage in India based on an estimation of R_0_. The developed model will help policymakers to take active measures prior to the further spread of COVID-19. Data on daily newly infected cases in India from March 2, 2020 to April 2, 2020 were to estimate R_0_ using the earlyR package. The maximum-likelihood approach was used to analyze the distribution of R_0_ values, and the bootstrap strategy was applied for resampling to identify the most likely R_0_ value. We estimated the median value of R_0_ to be 1.471 (95% confidence interval [CI], 1.351 to 1.592) and predicted that the new case count may reach 39,382 (95% CI, 34,300 to 47,351) in 30 days.

## INTRODUCTION

Coronavirus disease 2019 (COVID-19) has rapidly spread worldwide, with 896,450 confirmed total new cases and 45,526 deaths globally as of April 2, 2020 [1]. The disease emerged as 27 cases of pneumonia with an unknown cause in Wuhan, China. The first COVID-19 case in India was identified on January 30, 2020, and the total number of reported cases reached 2,322 as of April 3, 2020 [2]. On March 3, 2020, the Indian government suspended all new visas and visas issued to nationals of Iran, Italy, Japan, and Korea, and on the next day implemented compulsory screening of all international passengers. The Indian government declared a countrywide lockdown for 21 days on March 24, 2020 as a measure to control the spread of COVID-19, which has developed into a pandemic. The transmission rate of COVID-19 has been relatively low in most countries, but with major outbreaks in a few countries, such as Iran, Italy, Japan, and Korea. Most countries have at least an early stage of COVID-19 spread before any mitigation measures have an impact [3]. Myers et al. [4] stated that accurate epidemic forecasting models would noticeably improve epidemic prevention and control capabilities. No vaccine is available for COVID-19, and vaccination is typically not a good option for stopping the spread of a new epidemic, as considerable time is required to develop a safe and effective vaccine (approximately 10 years) [5]. Li et al. [6] found that the COVID-19 incubation period was 5.2 days (95% confidence interval [CI], 4.1 to 7.0) and found indications that human-to-human transmission occurred among close contacts. India is the second most populated country, it is important to estimate the transmissibility of COVID-19 and to predict the total number of new cases, which will help direct focus towards this public health crisis. Mathematically based epidemic models, such as susceptible-infected-recovered (SIR) models [7], susceptible-infected-susceptible (SIS) models [8], susceptible-exposed-infected-recovered (SEIR) models [9], and susceptible-exposed-infected-recovered-susceptible (SEIRS) models [10] are used to predict the trajectory of epidemics. Estimating the reproduction number (R_0_) can be estimated statistically or empirically. In this work, we used the earlyR (https://cran.r-project.org/) package to estimate R_0_ and predict the trajectory of the outbreak.

## METHODS

### Susceptible-exposed-infected-recovered-susceptible mathematical model

SEIR models can be used to predict the number of people infected based on R_0_. We have given a SEIR model in this study to demonstrate the importance of estimating R_0_ [11]. COVID-19 has an incubation period, also known as a latent period or latent delay (τ), of 2-14 days. The following assumptions were made for developing the mathematical model for COVID-19.

- The population growth of the region/country is exponential, and the COVID-19 epidemic is occurring in a sufficiently short period

- Infected individuals are assumed not to give birth

- Recovered individuals acquire permanent immunity with a probability *f*(0 ≤ f ≤ 1) or die from the disease with a probability of (1-*f*)

With S referring to susceptible individuals, E to susceptible individuals that become exposed at time t-τ, I to individuals who are infected, and R to those who have recovered from COVID-19, the resulting differential equations are:

(1) $$\frac{\mathrm{dS}(t)}{\mathrm{dt}}\;=\;b\;s(t)\;+\;bE(t)\;+\;bR(t)\;-\;\mu s(t)\;-\;\gamma \frac{I(t)\;S\;(t)}{N(t)}$$

(2) $$\frac{\mathrm{dE}(t)}{\mathrm{dt}}\;=\;\gamma \frac{I(t)\;S\;(t)}{N(t)}\;-\;\gamma \frac{I(t-\tau )\;S\;(t-\tau )}{N(t-\tau )}e^{-\mu \tau }\;-\;\mu \;E(t)$$

(3) $$\frac{\mathrm{dI}(t)}{\mathrm{dt}}\;=\;\gamma \frac{I(t-\tau )\;S\;(t-\tau )}{N(t-\tau )}e^{-\mu \tau }\;-\;\mu \;I(t)\;-\;\alpha \;I(t)$$

(4) $$\frac{\mathrm{dR}(t)}{\mathrm{dt}}\;=\;-\mu \;R(t)\;-\;f\;\alpha \;I(t)$$

Where μ is the per capita death rate due to causes other than the disease, γ is the rate of contact (or) transmission rate (or) infection rate, α is the recovery rate, and *b* is the per capita birth rate (with *b*>μ).

At any instant,

(5) S (t) + E (t) + I (t) + R(t) = N (t)

R_0_ is defined as,

(6) $$R_{0}\;=\;\frac{{\mathrm{\gamma e}}^{-\mathrm{b\tau}}}{b+\alpha }$$

This constant is extremely important in characterizing the spread of COVID-19. It reflects how many people contract the disease from an infectious individual. In general, If R_0_> 1, secondary infections will occur and the disease is spreading throughout the population. According to WHO information as of January 23, 2020, the R_0_ of COVID-19 lies between 1.4 and 2.5. R_0_ may vary considerably for different infectious diseases, but also for the same disease in different populations [12].

### Data

All the data shown in Table 1 were collected from an Indian official website [2]. The epidemiological data from March 2, 2020 to April 2, 2020, as shown in Table 1, were utilized to estimate R_0_. A higher R_0_ indicates a higher likelihood of new infections.

### Model development

The transmissibility of COVID-19 in India was evaluated using the earlyR package. It was assumed that interventions so far have had a minimal impact on COVID-19 transmission in India. The model used herein is a simplified version of the model introduced by Cori et al. [13]. Serial interval distributions (i.e., mean and standard deviation [SD]) are required to estimate R_0_. We assumed that the mean and SD were 4.7 days and 2.9 days, respectively, based on existing research [14]. The maximum-likelihood (ML) approach was applied to obtain the distribution of R_0_. The bootstrap strategy was applied for re-sampling 1,000 times to obtain likely R_0_ values. The R package projection was used to predict the cumulative daily incidence [15]. We forecast the cumulative total new cases after 30 days. The daily incidence obeys a Poisson distribution determined by daily infectiousness, which is denoted as,

(7) $$\lambda (t)\;=\;\sum_{k=1}^{t-1}X_{k}V(t-k)$$

Where V (t-k) the vector of the probability mass function and X_k_ is is the real-time incidence at time k. The forecasting model depended on the present incidence and serial interval distributions. The projections were based on resampling and probability computations. The statistical analysis and model development were done using R version 3.6.3 (https://cran.r-project.org/bin/windows/base/old/3.6.3/).

### Ethics statement

The analysis in the article is based on data which is open to public. The article does not require the ethical committee approval.

## RESULTS AND DISCUSSION

**Figure 1.**
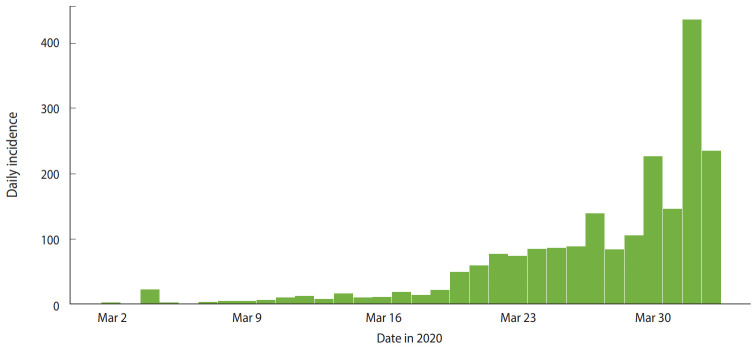
Actual daily incidence of coronavirus disease 2019 in India.

**Figure 2.**
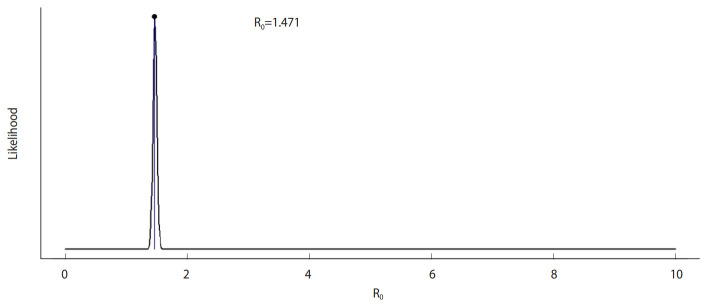
Maximum-likelihood value of reproduction number (R_0_).

**Figure 3.**
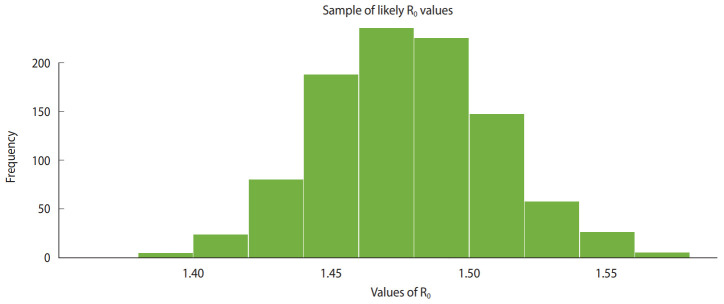
Sample of likely values of reproduction number (R_0_).

Figure 1 shows the daily incidence of COVID-19 in India from March 2, 2020 to April 2, 2020. Figure 2 shows the distribution of likely values of the R_0_ of COVID-19 in India. We estimated the ML value of R_0_ as 1.471 (95% CI, 1.351 to 1.592) for COVID-19 in the early stage in India. Figure 3 shows a histogram of R_0_ values using the bootstrap strategy with 1,000 likely samples.

**Figure 4.**
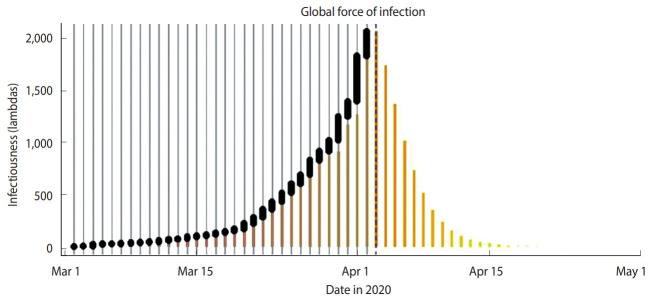
Global spread of infections.

**Figure 5.**
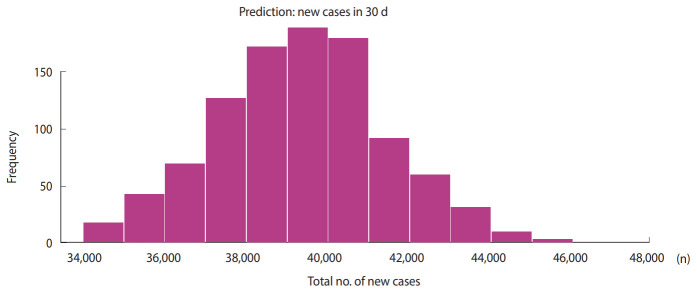
Predicted cumulative new cases in the next 30 days.

Figure 4 shows the global spread of COVID-19 during the same period. The vertical gray bars indicate the presence of cases and black dots denote the dates of symptom onset. The dashed vertical blue line indicates the current date (April 3, 2020). The vertical scale in Figure 4 shows the relative scale of infections. Figure 5 shows the predicted cumulative cases in next 30 days.

We computed that the cumulative number of new cases may reach 39,382 (95% CI, 34,300 to 47,351) in the next 30 days. The R_0_ data were estimated based on the existing COVID-19 data from March 2, 2020 to April 2, 2020. The Indian government has already announced a nationwide lockdown. As per the WHO information on January 23, 2020, the R_0_ of COVID-19 lies between 1.4 and 2.5. Our estimation indicates that for India, the median R_0_ value of 1.471 (95% CI, 1.351 to 1.592) is in the lower range. However, various studies have indicated that precisely estimating R_0_ is challenging, because R_0_ depends on environmental conditions, demography, and the modeling method. In our method, the accuracy of R_0_ depended on the premise that all cases of COVID-19 in India were identified in the study period. If the same scenario continues, we predict that the cumulative number of new cases may reach 39,382 (95% CI, 34,300 to 47,351) in next 30 days. We believe that our forecasting numbers may help in various aspects, such as developing the required medical infrastructure and focusing efforts on mitigating the economic impact of the pandemic. Our findings were derived based on a limited time frame, and the results may change after the occurrence of a considerable number of additional cases. The R_0_ value corresponding to the spread of COVID-19 can be controlled by strictly following social distancing in daily life, wearing masks, frequent hand-washing with soap or sanitizers, quarantining infected people, identifying cases using rapid diagnostic methods, and so on.

## CONCLUSION

We estimated the median value of R_0_ to be 1.471 (95% CI, 1.351 to 1.592) and predicted that the cumulative number of new cases may reach 39,382 (95% CI, 34,300 to 47,351) in the next 30 days. The predicted size largely depends on changes in R_0_. Effective measures against COVID-19 will help to reduce R_0_. The presence of numerous unidentified cases in the study period may result uncertainties in the estimated value of R_0_ used in the developed forecasting model.

## Figures and Tables

**Table 1.** Actual coronavirus disease 2019 daily new confirmed cases in India

Date in 2020	New confirmed cases (n)	Date in 2020	New confirmed cases (N)
Mar 2	2	Mar 18	14
Mar 3	1	Mar 19	22
Mar 4	22	Mar 20	50
Mar 5	2	Mar 21	60
Mar 6	1	Mar 22	77
Mar 7	3	Mar 23	74
Mar 8	5	Mar 24	85
Mar 9	5	Mar 25	87
Mar 10	6	Mar 26	88
Mar 11	10	Mar 27	140
Mar 12	13	Mar 28	84
Mar 13	8	Mar 29	106
Mar 14	16	Mar 30	227
Mar 15	10	Mar 31	146
Mar 16	11	Apr 1	437
Mar 17	19	Apr 2	235
